# The value of the infection prevention and control nurse led MRSA ward round

**DOI:** 10.1186/s13756-019-0506-6

**Published:** 2019-03-12

**Authors:** Mark I. Garvey, Craig W. Bradley, Martyn A. C. Wilkinson, Kerry L. Holden, Victoria Clewer, Elisabeth Holden

**Affiliations:** 1 University Hospitals Birmingham NHS Foundation Trust, Queen Elizabeth Hospital Birmingham, Edgbaston, Birmingham, B15 2WB UK; 2 Institute of Microbiology and Infection, The University of Birmingham, Edgbaston, Birmingham, UK; 3 Gloucestershire Hospitals NHS Foundation Trust, Gloucestershire Royal Hospital, Gloucester, GL1 3NN UK

**Keywords:** MRSA bacteraemia’s, MRSA, Infection control nurse led ward rounds

## Abstract

Meticillin-resistant *S. aureus* (MRSA) is prevalent in most parts of the world. The study took place at Queen Elizabeth Hospital Birmingham (QEHB) a UK tertiary referral hospital. At QEHB innovative nurse led daily ward rounds for patients that acquire hospital acquired MRSA during their hospital stay are undertaken. The aim is to optimise care delivered for these patients whilst at QEHB, thereby reducing the risk of infection in patients with healthcare-acquired MRSA. A segmented Poisson regression model suggests that the MRSA bacteraemia rate was affected where an 88.94% reduction (*p* = 0.0561) in bacteraemias was seen by the introduction of these ward rounds. We describe a nurse led MRSA ward round which was associated with a lower rate of MRSA bacteraemias.

## Background

*Staphylococcus aureus* is a major cause of healthcare-associated infection worldwide [1–3]. Meticillin-resistant *S. aureus* (MRSA) has become prevalent in most parts of the world [1–3]. Despite its decline in incidence in several European countries, MRSA infection remains a major cause of avoidable morbidity and mortality in patients admitted to hospital [1–3]. It results in increased length of hospital stay, risk of death and treatment costs, with colonised and infected patients acting as reservoirs for the spread of MRSA within hospitals [1–3]. Isolation and decolonisation are the two main targeted control measures for reducing transmission [2, 3].

Bradley et al.*,* (2017) previously reported the effect of universal MRSA decolonisation therapy in Intensive Care Unit (ICU) patients regardless of patients’ MRSA status [2]. A breakpoint model was used to identify an increase in MRSA bacteraemias and acquisitions across QEHB after the withdrawal of universal decolonisation [2]. As a result, universal decolonisation was reintroduced into the ICU resulting in a reduction of MRSA bacteraemias and acquisitions across QEHB [2]. Garvey et al.*,* (2018) followed on from this work and used a Poisson model to demonstrate that the average hospital acquisition rate of MRSA/100,000 patient bed days reduced by 6.3% per month after the introduction of a new universal wipe regime [3]. Here, we follow on from both these MRSA reduction strategies, by detailing the effect of introducing a nurse-led ward round of patients with healthcare associated MRSA while inpatients at QEHB. Over the past 2 years all the MRSA bacteraemias apportioned to QEHB were from patients whom acquired hospital acquired MRSA while inpatients. As a response to this QEHB introduced a nurse-led ward round specifically focusing on this patient cohort.

## Methods and materials

### Setting

QEHB, part of University Hospitals Birmingham (UHB) NHS Foundation Trust is a tertiary referral teaching hospital in Birmingham, UK that provides clinical services to nearly one million patients every year [2, 3].

### MRSA screening

MRSA screening of all emergency and elective surgical patients admitted to UHB is standard practice. Swabs are taken from the nose, groin and throat as well as any wounds or sites of invasive devices. Inpatients with greater than 28 days’ stay are rescreened every 4 weeks. MRSA screens and clinical specimens were cultured as described previously on Chromagenic agar [1–3].

### MRSA acquisitions

At QEHB, a patient is defined as acquiring MRSA if they have a negative admission screen and then have MRSA isolated from a subsequent screen or clinical specimen, 48 h or more after admission. Only MRSA acquisitions at QEHB were included in the analysis [2, 3].

### MRSA bacteraemias

All MRSA bacteraemias were attributed as healthcare associated or non-healthcare associated using the criteria of the Centres for Disease Control and Prevention/National Healthcare Safety Network for national reporting purposes [4]. Only bacteraemias that were assigned to QEHB were included in the analysis [2, 3].

### Holistic nurse led MRSA ward round

From January 2017 onwards, QEHB commenced an innovative Infection Prevention and Control nurse (IPCN) led daily MRSA ward rounds on patients that acquire hospital acquired MRSA during their hospital stay. The aim was to optimise care delivered for these patients whilst in hospital, thereby reducing their risk of developing infection. Patients are assessed by IPCNs daily for clinical features of infection. For example raised inflammatory markers such as C-reactive protein and white cell count, signs of sepsis including increase in temperature, change in blood pressure and respiratory rate, other signs of infection including purulent wounds or erythema to name but a few. There were no general criteria just clinical judgement looking whether a patient had an infection or not. Patients are seen at the bedside and physiological parameters and laboratory markers are reviewed. When signs of infection are present, these cases are escalated to medical staff. In addition, patients are assessed for the presence of invasive devices, for example urinary catheters, peripheral or central venous catheters and dialysis catheters, as well as for the presence of invasive ventilation. All devices are reviewed based on national guidance and removed if no longer required [5]. Wound and skin assessments are undertaken to identify breaks in the skin and/or the presence of skin conditions. Patients’ decolonisation therapy is checked to ensure compliance with QEHB’s policy for decolonisation and correct application technique [2]. The ward rounds include confirmation that appropriate Infection Prevention and Control precautions are in place, including isolation, hand hygiene compliance and the correct use of personal protective equipment such as gloves and aprons. The antimicrobial prescriptions of these patients are also reviewed to ensure there is appropriate MRSA cover if bacterial infection is suspected, with any concerns/queries being escalated to clinicians and the clinical microbiology service.

### Statistical analysis

Segmented Poisson regression models containing offsets for patient bed days were used to detect any significant changes in the rate of MRSA acquisitions and bacteraemias from May 2016 – November 2018. This date range was chosen to specifically analyse the effect of the MRSA ward round which was implemented in January 2017 [2, 3]. ‘Full’ models were constructed containing terms for changes in level and gradient associated with the following potentially-important intervention: the implementation of holistic MRSA acquisition ward rounds. Backward step-wise regression was performed on the ‘full’ Poisson models, with the Akaike Information Criterion used to select the models that best fit the data, whilst guarding against over-fitting. All statistical analyses were performed using R version 3.4.3 [6, 7].

### Audits

Regular audits were undertaken by the Infection Prevention and Control Team during the period of this report. Audits included monitoring hand hygiene compliance; monitoring the appropriate use of personal protective equipment and monitoring environmental cleanliness.

## Results

### MRSA acquisitions and bacteraemias

**Fig. 1:**
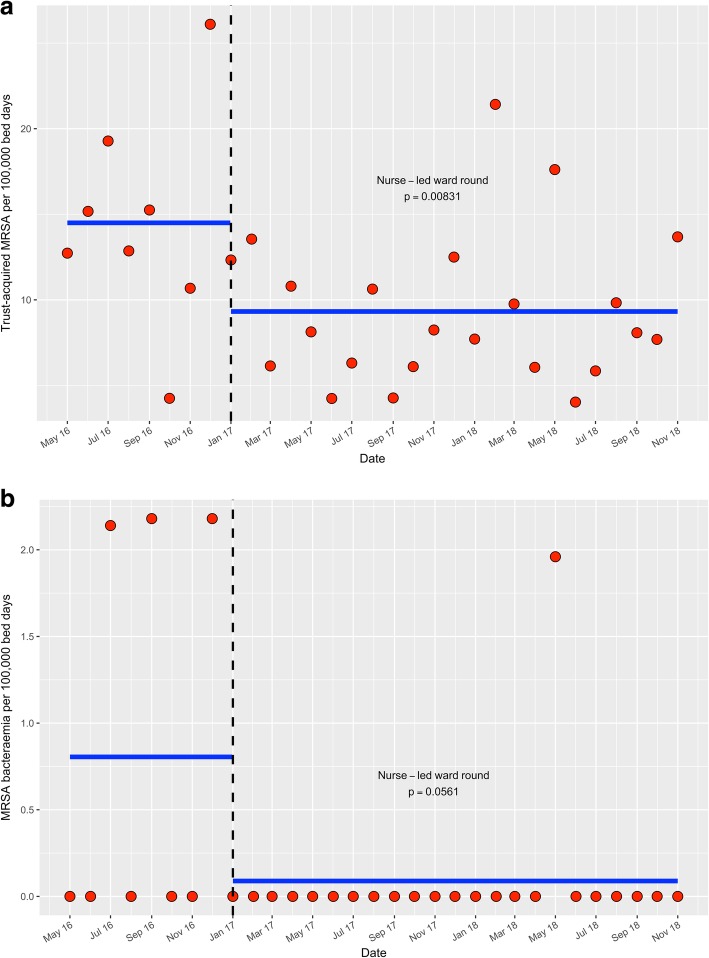
Using a segmented Poisson regression model changes in hospital wide monthly MRSA acquisition (**a**) and bacteraemia rates (**b**) per 100,000 bed days between May 2016–November 2018. Key: The dotted lines represent the infection prevention and control interventions. The blue lines represent the mean values predicted by the Poisson regression model

The optimum segmented Poisson models selected contained terms for a change in the level of acquisitions and bacteraemias, but no trend terms; the models suggest that the number of MRSA acquisitions and bacteraemias were immediately affected by the introduction of the MRSA acquisition nurse led ward round (Fig. 1a and b). The model shows, for MRSA acquisitions, there was a reduction in mean from 14.50 per 100,000 bed days between May 2016–December 2016, to 9.32 per 100,000 bed days between January 2017–November 2018 (Fig. 1a). This was a 35.7% reduction (*p* = 0.008, 95% confidence interval = (10.71, 53.70%)). The model also demonstrates for MRSA bacteraemias, there was a reduction in mean from 0.805 per 100,000 bed days between May 2016 – December 2016, to 0.089 per 100,000 bed days between January 2017 – November 2018 (Fig. 1b). This was an 88.94% reduction (*p* = 0.0561, 95% confidence interval = (− 6.38, 98.85%)).

### Auditing

Audits revealed no significant changes in hand hygiene compliance; appropriate use of personal protective equipment or environmental cleanliness during these periods (data not shown). MRSA screening for emergency and elective patients averages never changed throughout the study periods. At UHB there is electronic prescribing so all patients whom are MRSA positive receive decolonization therapy.

## Discussion

Between May 2016 to November 2018, all MRSA bacteraemias apportioned to QEHB were in patients who had acquired MRSA as an inpatient. At QEHB, patients who acquire MRSA while in hospital were therefore identified to be at highest risk of developing MRSA bacteraemias. This is not unexpected, since Torok et al.*,* (2014) previously detailed in their hepatology population, higher MRSA acquisition rate is often associated with higher MRSA bacteraemias [8]. Roghmann et al.*,* 2001 also demonstrated a statistically significant high-risk ratio in patients with MRSA bacteraemias when there was MRSA colonisation of their chronic wounds [9]. There are numerous reports in the literature describing MRSA acquisition and the heightened risk of MRSA bacteraemias, as seen in the QEHB bacteraemia patient group [8, 9].

Due to the fact that patients acquiring MRSA at QEHB are at greatest risk of developing an MRSA bacteraemia, QEHB introduced a daily IPCN nurse led ward round specifically focusing on this patient cohort. A segmented Poisson regression model suggests that the rate of MRSA acquisitions and bacteraemias have diminished since introduction of the MRSA acquisition ward rounds. Overall, there is good evidence that the introduction of a holistic nurse led ward MRSA acquisition round was associated with a reduction in trust apportioned acquisitions of MRSA, as well as some weaker evidence of a concomitant decrease in bacteraemias. Whilst the *p*-value of 0.0561 associated with the reduction in bacteraemias falls a little short of the value usually regarded as formally significant, we feel that the decrease is important, and that further data will underline its significance. Traditional power calculations predict that the difference in the mean bacteraemias of 0.716, which comprises a nearly 90% reduction, would be detected with a power of approximately 85% when the level of significance is set at 5%. However, these calculations assume heterogeneity of variance, which is clearly not the case with MRSA bacteraemias; such events are much rarer after the introduction of the ward rounds. There is a wealth of literature describing successful interventions from nurse led wards rounds. Brink et al.*,* (2016) discussed the role of pharmacists and nurses in the reduction of antimicrobial consumption [10]. Catangui et al.*,* (2012) previously described the benefits of a nurse led ward round in acute stroke care [11]. Bradley et al.*,* (2018) saw lower rates of recurrent *Clostridium difficile* infection and reduced length of stay as a result of specialist IPCN led *C. difficile* ward rounds [11]. IPCN led ward rounds on various nosocomial infections and clinical syndromes have the potential to impact on the basics of patient care for the better.

It is important to note that there are confounders to this observation that nurse led ward rounds have resulted in a reduction in the number of MRSA bacteraemias seen at QEHB. Firstly, as per Bradley et al.*,* (2017), universal decolonisation of ICU patients has resulted primarily in a reduction in the number of MRSA bacteraemias, so this could affect the results seen in this report [2]. Secondly, as a result of the alteration in cleaning as per Garvey et al.*,* (2018), the change in practice at QEHB from a two wipe system, to a universal one wipe cleaning regime has resulted in a reduction of the number of MRSA acquisitions across QEHB [3]. This observation will have an effect on the results seen in this report, as the number of MRSA acquisitions have decreased, thereby there are less patients acquiring MRSA to go on to develop an MRSA bacteraemia. It is of note that the acquisitions seem to have decreased after the introduction of the ward rounds. It could be that these rounds have reduced transmission between MRSA positive patients and the general hospital population, maybe by decreasing environmental shedding.

## Conclusions

Here, we describe some novel interventions in our local management of MRSA, which have accounted for a lower rate of MRSA bacteraemias. We suggest nurse led MRSA acquisition ward rounds should be undertaken in clinical practice to prevent MRSA acquisitions progressing to MSRA bacteraemias. These successful nurse led MRSA ward rounds could be adapted and utilised for other nosocomial alert organisms such as multi-drug resistant organisms, with the potential to observe better patient outcomes.

## Abbreviations

ICU: Intensive care unit
IPCN: Infection Prevention and Control Nurse
MRSA: Meticillin-resistant *S. aureus*
QEHB: Queen Elizabeth Hospitals Birmingham
UHB: University Hospitals Birmingham
